# Transcriptome microRNA profiling of bovine mammary epithelial cells challenged with *Escherichia coli* or *Staphylococcus aureus* bacteria reveals pathogen directed microRNA expression profiles

**DOI:** 10.1186/1471-2164-15-181

**Published:** 2014-03-07

**Authors:** Weiwu Jin, Eveline M Ibeagha-Awemu, Guanxiang Liang, Frédéric Beaudoin, Xin Zhao, Le Luo Guan

**Affiliations:** 1 Department of Agricultural, Food and Nutritional Science, University of Alberta, Edmonton, AB T6G2P5, Canada; 2 Agriculture and Agri-Food Canada, Dairy and Swine Research and Development Centre, 2000 College Street, Sherbrooke, QC J1M 0C8, Canada; 3 Department of Animal Science, McGill University, 21111 Lakeshore Road, Ste-Anne-De-Bellevue, QC H9S 3V9, Canada

**Keywords:** microRNA, *E. coli*, *S. aureus*, Mastitis, MicroRNA-sequencing, Bovine mammary epithelial cells

## Abstract

**Background:**

MicroRNAs (miRNAs) can post-transcriptionally regulate gene expression and have been shown to be critical regulators to the fine-tuning of epithelial immune responses. However, the role of miRNAs in bovine responses to *E. coli* and *S. aureus,* two mastitis causing pathogens, is not well understood.

**Results:**

The global expression of miRNAs in bovine mammary epithelial cells (MAC-T cells) challenged with and without heat-inactivated *Staphylococcus aureus (S. aureus)* or *Escherichia coli (E. coli)* bacteria at 0, 6, 12, 24, and 48 hr was profiled using RNA-Seq. A total of 231 known bovine miRNAs were identified with more than 10 counts per million in at least one of 13 libraries and 5 miRNAs including bta-miR-21-5p, miR-27b, miR-22-3p, miR-184 and let-7f represented more than 50% of the abundance. One hundred and thirteen novel miRNAs were also identified and more than one third of them belong to the bta-miR-2284 family. Seventeen miRNAs were significantly (P < 0.05) differentially regulated by the presence of pathogens. *E. coli* initiated an earlier regulation of miRNAs (6 miRNAs differentially regulated within the first 6 hrs post challenge as compared to 1 miRNA for *S. aureus*) while *S. aureus* presented a delayed response. Five differentially expressed miRNAs (bta-miR-184, miR-24-3p, miR-148, miR-486 and let-7a-5p) were unique to *E. coli* while four (bta-miR-2339, miR-499, miR-23a and miR-99b) were unique to *S. aureus*. In addition, our study revealed a temporal differential regulation of five miRNAs (bta-miR-193a-3p, miR-423-5p, miR-30b-5p, miR-29c and miR-un116) in unchallenged cells. Target gene predictions of pathogen differentially expressed miRNAs indicate a significant enrichment in gene ontology functional categories in development/cellular processes, biological regulation as well as cell growth and death. Furthermore, target genes were significantly enriched in several KEGG pathways including immune system, signal transduction, cellular process, nervous system, development and human diseases.

**Conclusion:**

Using next-generation sequencing, our study identified a pathogen directed differential regulation of miRNAs in MAC-T cells with roles in immunity and development. Our study provides a further confirmation of the involvement of mammary epithelia cells in contributing to the immune response to infecting pathogens and suggests the potential of miRNAs to serve as biomarkers for diagnosis and development of control measures.

## Background

Mastitis is an infectious disease of the mammary gland that has been considered as one of the most economically important diseases in the dairy industry for several decades [1]. Mastitis can be caused by a variety of bacteria including Gram-negative *Escherichia coli* (*E. coli*) and Gram-positive *Staphyloccucus aureus* (*S. aureus*) [2]. The mastitis caused by *E. coli* and other Gram-negative bacteria is often clinical with an acute and severe inflammation and the pathogens may be eventually cleared by the immune system within days or with antibiotic treatment [3,4]. In contrast, infection with Gram-positive bacteria like *S. aureus* often causes mild mastitis and the clearance of the pathogens by antibiotics is often ineffective [5].

Efforts at mastitis control include understanding host response mechanisms to infecting pathogens and development of appropriate control strategies. Transcriptomic as well as proteomic profilings have shown a marked difference in the response of host to *E. coli* and *S. aureus* bacteria. Transcriptomic studies on bovine mammary epithelial cells *in vitro* challenged with mastitis pathogens and/or mammary gland tissues collected after intramammary infection revealed very different mechanisms in host innate immune responses to pathogens [6-8]. Differential cytokines and chemokines (at protein level) and other immune response proteins were also observed in bovine mammary epithelial cells and tissues or milk, in response to *E. coli* and *S. aureus*[4,9,10].

Recently, an endogenous noncoding small RNA known as microRNA (miRNA) has been shown to be involved in a wide variety of biological processes such as development, differentiation, apoptosis and viral infection. The role of miRNA in the regulation of the innate and acquired immune response has been well reviewed [11-14]. It was shown that miR-155 promotes the production of TNF-α in human embryonic kidney cells (HEK-293), suggesting the positive role of miR-155 to modulate the release of inflammatory mediators [15]. Recent studies on human epithelial cells infected by a protozoan parasite (*Cryptosporidium parvum*) revealed the up-regulation of let-7i or miR-27b. While let-7i directly regulates the expression of toll-like receptor 4 (TLR4) [16], miR-27b targets KH-type splicing regulatory protein (KSRP) to coordinate TLR4-mediated epithelial immune responses to pathogens [17], indicating that miRNA may act as a critical regulator in epithelial immune responses. In addition, it was suggested that miRNA may modulate epithelial immune responses at every step of the innate immune network, including production and release of cytokines/chemokines, expression of adhesion and costimulatory molecules [18]. It has been clearly demonstrated that bovine mammary epithelial cells mount a robust immune response to the presence of bacterial pathogens [19-21]. However, how miRNAs modulate this robust immune response by the bovine mammary epithelial cells to the presence of the mastitis pathogens, *E. coli* and *S. aureus* bacteria, is poorly understood. Furthermore, studies elucidating the regulatory roles of miRNA in bovine immunity and infection are few. A recent study using quantitative real time PCR technique revealed differential expression of five inflammation related miRNAs (miR-9, miR-125b, miR-155, miR-146a and miR-223) after stimulation of bovine monocytes with lipopolysaccharide (LPS) and *S. aureus* enterotoxin B [22]. Using the same technique, four miRNAs (bta-miR-181a, miR-16, miR-31 and miR-223) out of 14 miRNAs associated with regulation of innate immunity and mammary cell function were shown to be differentially regulated in bovine mammary tissue challenged with *Streptococcus uberis* (*S. uberis*) [23]. These studies suggest roles of miRNAs in bovine mammary gland immunity but the extent of information is limited by the technique used. Increasingly, more robust techniques like microarray and next-generation sequencing are finding applications in the elucidation of miRNAs in immunity [24,25]. In particular, next-generation sequencing has the ability to profile the expression of both known and novel miRNAs with high resolution and accuracy and to distinguish miRNAs that are very similar in sequence as well as isomiRs [26]. Only two studies to date have used next-generation RNA sequencing to study the regulatory roles of miRNAs upon bacterial or viral infections in bovine [25,27]. Using RNA sequencing, 21 miRNAs were detected as significantly differentially expressed upon infection of bovine primary epithelial cells with *S. uberis,* as well as a unique miRNA profile in response to a Gram-positive bacterial infection [25]. However, more information is needed to understand the roles of miRNAs in modulating bovine mammary gland infections for their effective application as biomarkers of mastitis or as therapeutic agents.

To investigate the role of miRNA in host defense to two mastitis pathogens, miRNA expression in bovine mammary epithelial cells (MAC-T) challenged with heat-inactivated Gram-negative *E. coli* stain P4 or Gram-positive *S. aureus* strain Smith CP bacteria were characterized by next-generation sequencing at different time points (6, 12, 24 or 48 hr) following challenge and at 0, 6, 12, 24 or 48 hr without challenge. The next-generation sequencing technology allowed simultaneous detection of known and novel bovine miRNAs, and global miRNAs expression as well as pathogen directed differential miRNA expression patterns.

## Results

### miRNA sequencing

**Figure 1:**
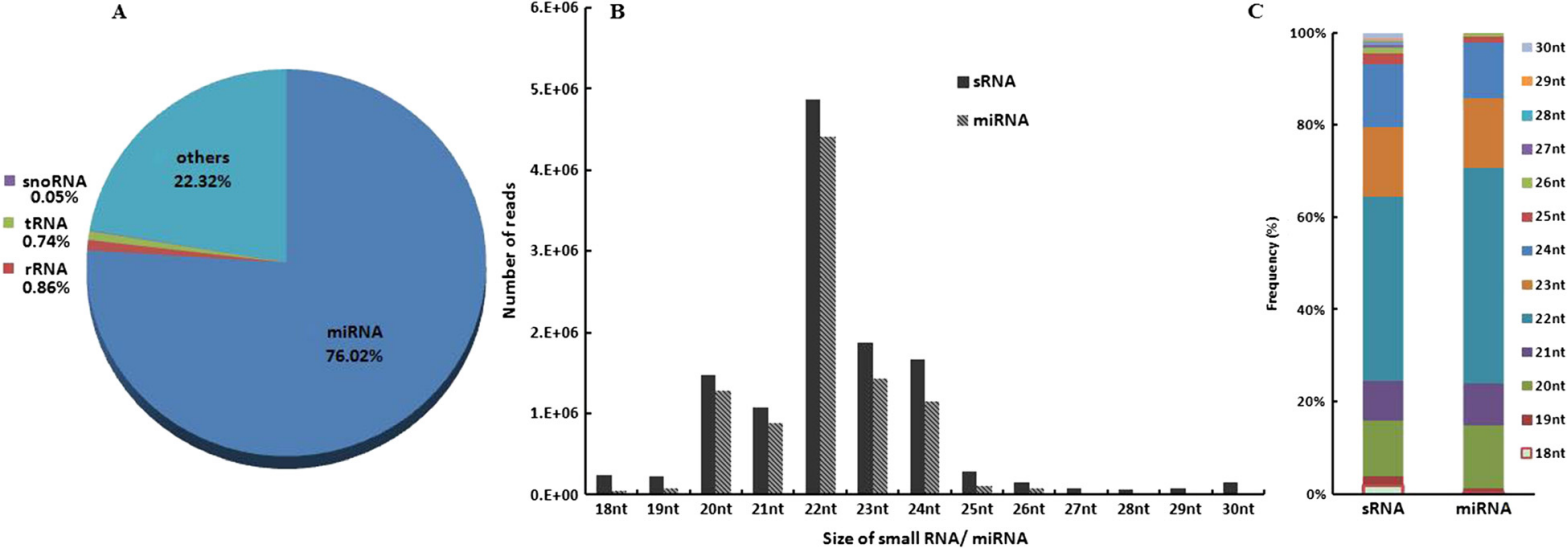
**Detected small RNAs.** A pie graph showing the relative abundance of different classes of small RNAs **(A)**; size distribution of small RNAs or miRNAs **(B)**; frequency distribution of each size (18–30 nts) of small RNAs or all known miRNAs based on all reads **(C)**. miRNA here only indicates known bovine miRNAs in miRBase (Release 19).

Thirteen small RNA libraries were constructed and sequenced simultaneously. A total of 15,315,312 high-quality reads were generated. Among them, 12,272,208 sequences ranging from 18 to 30 nucleotides were obtained after adaptor trimming, accounting for 80.1% of all small RNA (sRNA) sequences. Alignment with miRBase (Release 19) revealed that miRNAs were highly enriched in all libraries. Of the 18 to 30 nucleotide sRNA fraction, more than three quarters (76.02%) of them were identified as known bovine miRNAs, while only a small number (< 2%) aligned to bovine tRNAs, rRNAs and snoRNAs. The remaining reads were other sRNAs including novel bovine miRNAs (~0.2%), loop sequences of miRNA precursors and sequencing artifacts (Figure 1A). The majority of reads from sRNAs and known miRNAs were 20 to 24 nucleotides in length (comprising 89.4% and 96.6% of their total number, respectively). Dominant reads of sRNAs or known miRNAs were 22 nucleotides in length (~50%), followed by 23, 24 or 20 nucleotides, and lastly 21 nucleotides (Figure 1B, C).

In total, we identified 231 known bovine miRNAs with more than 10 counts per million (CPM) in at least one library. Among them, 18 highly expressed miRNAs accounted for 78.55% of the total reads of identified known miRNAs (Table 1). The top five highly expressed miRNAs were bta-miR-21-5p, miR-27b, miR-22-3p, miR-184 and let-7f, accounting for 16.02%, 15.18%, 8.37%, 5.45% and 5.01% of total known miRNA reads, respectively.

**Table 1:** **Top expressed miRNAs in bovine MAC-T cells with or without challenge with *E. coli *** **or *S. aureus *** **bacteria**

**miRNA ID**	**Total no. of reads**	Ratio*			
		**All samples**	**Control group**	***E. coli *** **group**	***S. aureus *** **group**
bta-miR-21-5p	1494737	16.02%	15.89%	16.18%	16.07%
bta-miR-27b	1416234	15.18%	14.65%	15.46%	15.77%
bta-miR-22-3p	780733	8.37%	8.61%	8.38%	7.95%
bta-miR-184	508224	5.45%	5.65%	5.27%	5.30%
bta-let-7f	467126	5.01%	4.83%	4.94%	5.39%
bta-miR-205	424274	4.55%	4.56%	4.67%	4.39%
bta-miR-92a	363202	3.89%	3.74%	4.02%	4.01%
bta-miR-182	362632	3.89%	3.60%	3.87%	4.41%
bta-miR-31	261303	2.80%	2.92%	2.80%	2.60%
bta-let-7a-5p	190668	2.04%	2.19%	1.81%	2.05%
bta-miR-222	172392	1.85%	1.88%	1.84%	1.80%
bta-let-7i	166440	1.78%	1.73%	1.75%	1.92%
bta-miR-221	141821	1.52%	1.57%	1.51%	1.44%
bta-miR-26a	129420	1.39%	1.45%	1.39%	1.28%
bta-miR-148a	123204	1.32%	1.24%	1.40%	1.37%
bta-miR-27a-3p	118012	1.26%	1.34%	1.26%	1.14%
bta-miR-186	104935	1.12%	1.12%	1.15%	1.10%
bta-miR-191	103301	1.11%	1.08%	1.13%	1.12%
Total	7328656	78.55%	78.04%	78.84%	79.11%

*Ratio refers to the total number of reads of a miRNA as compared to all reads of known bovine miRNAs detected in this study (miRBase Release 19).

### Identification of novel miRNAs and miRNA candidates

With the high-throughput sequencing data, miRDeep2 [28] generated a score for each known miRNA and novel miRNA. The score of 5.0 yielded a signal-to-noise ratio of 16.2 [28] and was used as cut-off for novel miRNA prediction (Additional file 1: Table S1.1). A total of 114 novel miRNA hairpins were identified (Additional file 2: Table S2). Moreover, 186 hairpins with lower scores (0–4.9) but having more than 10 total counts were further analyzed and 5 or 153 of them were defined as precursors of novel miRNAs or miRNA candidates, respectively (Additional file 3: Table S3). Although almost all miRNAs have a variety of isoforms, most of the known miRNAs detected or undetected by miRDeep2 (Additional file 1: Table S1.2 and Additional file 1: Table S1.3) were within the size range of 20 to 24 nucleotides. Moreover, a false precursor of bta-miR-532 predicted by miRDeep2 (Additional file 1: Table S1.2) contained a star miRNA of 17 nucleotides in length, indicating that a precursor generating a short miRNA may have a high false positive rate. Therefore, precursors which form mature miRNAs < 20 nucleotides in length were not further analyzed.

In total, five homologs of known miRNA precursors from other animal species and ten reversely complementary to known bovine miRNA precursors were identified to generate 24 novel miRNAs (Table 2, Additional file 2: Table S2 and Additional file 3: Table S3). We also found an ortholog of bta-miR-222 (bta-miR-222b, Table 2, Additional file 3: Table S3). In addition, more than one third of novel or candidate miRNA precursors (41/114, 65/153, respectively) were found to be clustered together and have a high similarity to those of bta-mir-2284 family in sequences (Additional file 2: Table S2, Additional file 3: Table S3 and Additional file 4: Figure S1). The remaining 62 novel miRNA precursors were found to produce 56 mature miRNAs which are less conserved to any known miRNA in miRBase (Release 19, Additional file 2: Table S2).

**Table 2:** **A partial list of novel miRNAs identified in bovine MAC-T cells with or without challenge with *E. coli or S. aureus *** **bacteria**

**Mature miRNA ID**^**1**^	**miRBase Pre-miRNA**^**2**^	**miRBase miRNA**^**3**^	^**5**^**Score**	**Mature reads**	**Mature sequence**	**Size**	**Star reads**	**Star sequence**^**4**^	**Size**	**Precursor coordinate**
**bta-miR-1842-5p**	eca-mir-1842	eca-miR-1842	1180.4	2,172	uggcucugugaggucggcuca	21	142	agcaggccugucagggcguug	21	25:1744660..1744720:-
**bta-miR-219-2-3p**	ssc-mir-219	ssc-miR-219	68.9	103	agaguugagucuggacgucccg	22	20	ugauuguccaaacgcaauuc	20	23:7335349..7335412:-
**bta-miR-664-3p**	ssc-mir-664	ssc-miR-664-3p	68.5	70	uauucauuuaucucccagccuaca	24	59	caggcuaggagaaaugauuggau	23	16:24379875..24379936:-
**bta-miR-6516-5p**	gga-mir-6516		9.6	10	uuugcaguaacaggugugaac	21	9	aucauguaugauacugcaaaca	22	19:55419754..55419817:+
**bta-mir-2964a-3p**	bta-mir-219-1′	hsa-miR-2964a-3p	20.7	27	agaauugcguuuggacaaucagu	23	5	gauguccagccacaauucucg	21	11:98997166..98997230:+
**bta-miR-3548-3p**	bta-mir-200a′	rno-miR-3548	6.6	2	cagcacuguccgguaagauguc	22	2	caucguuaccagacaguguuaga	23	16:52521338..52521399:+
**bta-miR-194-1′-3p**	bta-mir-194-1′		30.4	56	acauggaguugcuguuacaaucg	23	3	aaguaacagcaucuccacugga	22	16:24306044..24306105:+
**bta-miR-486′-5p**	bta-mir-486′		27.8	48	cccgguacugagcugacccgag	22	4	ucggggcagcucaguacaggac	22	27:36261849..36261912:+
**bta-miR-374a′-5p**	bta-mir-374a′	bta-miR-374a-3p	26.1	50	uuacaauacaaccugauaagug	22	1	acuuaucagguuguauuaua	20	X:81951234..81951283:-
**bta-miR-763′-3p**	bta-mir-763′		6.8	8	ucccagcuggucauuaauccu	21	3	gaauuaauggcuggcugggagg	22	5:48164035..48164101:+
**bta-miR-191′-5p**	bta-mir-191′	bta-miR-191	5.5	40	gaacgaaauccaagcgcagcug	22	0	gcugcuuuugggauuccguugcc	23	22:51543481..51543545:-
**bta-miR-1388′-3p**	bta-mir-1388′	bta-miR-1388-5p	1.8	405	uucucagguuggacaguccuga	22	0	cgggcugacaaaccugagauug	22	13:54375941..54376002:-
**bta-miR-374b′-5p**	bta-mir-374b′	bta-miR-374-3p	1.4	60	ugauaauacaaccugauaagug	22	0	cuuagcagguuguauuauaucc	22	X:82023929..82023981:-
**bta-miR-194-2′-3p**	bta-mir-194-2′	bta-miR-194	4.8	10	cacauggaguugcuguuaca	20	0	uaacagcagccccacugga	19	29:43731473..43731526:-
**bta-miR-590-3p**	eca-mir-590		1.4	13	uaguuuuauguguaagcucguu	22	0	cgagcuuauucacagaaguaca	22	25:33713356..33713419:-
**bta-miR-222b-3p**		bta-miR-222	4.9	20	ugcuacaucuggcuacugga	20	0	ggguggacgcuguguggcugagc	23	10:78769129..78769183:+

^1^“`”represents the antisense of miRNA precursor; the star sequence of miR-219-2 is miR-219-5p in miRBase (Release 19);

^2^The top known miRNA precursor (miRBase Release 19) aligns to novel precursor with > 70 identity and > 75 sequence length;

^3^The top known miRNA (miRBase Release 19) matches novel miRNA covering > 90 identity and > 90 sequence length;

^4^Sequence here only shows the dominant one and the star sequence that was absent was predicted by miRDeep2.

^5^miRNA with score less than 5 is identified from miRNA candidate precursor.

### Time effect on miRNA expression in MAC-T cells

**Figure 2:**
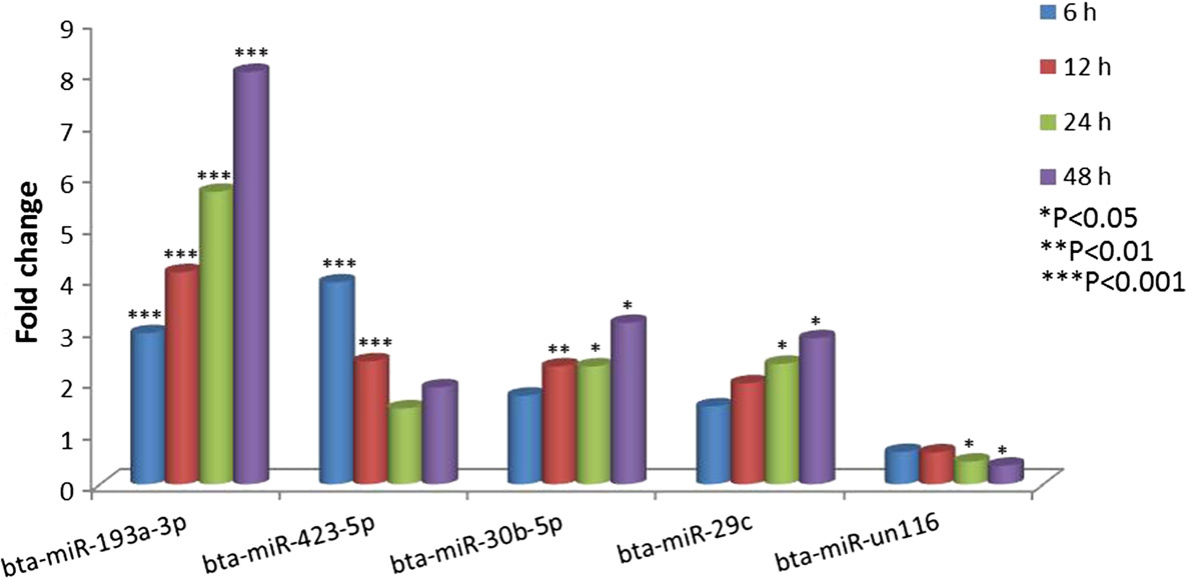
Five differentially expressed miRNAs in control cells over time as compared to 0 hr.

Considering that miRNAs play roles in almost all biological processes, we hypothesized that the expression of miRNAs might be regulated during the 48 hr incubation period of MAC-T cells with or without pathogenic bacteria. The global expression of miRNAs at different time points and under treatments were profiled and the correlations between libraries were performed with the normalized counts of all detected miRNAs (CPM >10 in at least one library). The correlations between libraries were very high (Additional file 5: Figure S2), suggesting that only a limited number of miRNAs might be significantly regulated during the 48 hrs of cell challenge with mastitis pathogens. The lowest correlation was found between 0 hr and 48 hrs in control samples (R^2^ = 0.961), indicating that the expression of miRNA might be significantly regulated over time. Therefore, differentially expressed miRNAs between different time points and 0 hr in control cells were analyzed using DEseq, an R/Bioconductor package [29] method to compare differential expression based on sequence counts. We observed that five miRNAs (bta-miR-193a-3p, miR-423-5p, miR-30b-5p, miR-29c and miR-un116) were differentially expressed (P < 0.05) in at least two time points in control cells as compared to 0 hr (Figure 2). The expression of bta-miR-193a-3p was the most regulated over time, increasing lineally and reaching 8.03 fold increase (P < 0.0001) within 48 hrs of cell growth. The expression of bta-miR-30b-5p and bta-miR-29c also increased over time but to a lesser magnitude as compared to bta-miR193a-3p. The expression of bta-miR-423-5p was highest at 6 hrs (P < 0.001) and returned to 0 hr level by 24 hrs (Figure 2). Conversely, the expression of bta-miR-423-5p and bta-miR-un116 decreased with time. The expression of bta-miR-193a-3p and bta-miR-423-5p in control cells was further validated by qRT-PCR. Both miRNAs were significantly up-regulated and reached a peak at 12 hrs, although no change larger than 2-fold was found between any two time points (Additional file 6: Figure S3).

### Effect of pathogenic *E. coli or S. aureus* bacteria on miRNA expression in MAC-T cells

To determine the effect of *E. coli* and *S. aureus* bacteria on miRNA expression in MAC-T cells during the 48 hr incubation period, we compared the expression of miRNAs at each time point to control samples at the same time point. The expression of 17 miRNAs were differentially affected (Table 3) by the presence of pathogenic bacteria in MAC-T cells. Within 6 hrs of the presence of *E. coli*, the expression of 6 miRNAs in MAC-T cells was significantly altered (P < 0.05), three were down regulated (bta-miR-193a-3p, miR-30c and miR-30b-5p) while three were up-regulated (bta-miR-365-3p, miR-184 and miR-24-3p) (Table 3). With time, the number of differentially expressed miRNAs in the presence of *E. coli* decreased and by 48 hrs, only two miRNAs were significantly altered. Conversely in *S. aureus* challenged cells, only one miRNA, bta-miR-2339, responded significantly to the presence of *S. aureus* bacteria in the first 6 hrs by showing a 4.56 fold up regulation (P < 0.05) as compared to control cells. Furthermore, the expression of more miRNAs became affected by the presence of *S. aureus* with increasing time (4 miRNAs at 16 hrs, 5 at 24 hrs and 4 at 48 hrs). The three miRNAs (bta-miR-193a-3p, miR-30c and miR-30b-5p) that were significantly down regulated or one miRNA (bta-miR-365-3p) that was significantly up regulated within 6 hrs of *E. coli* presence only showed a retarded significant down regulation by 24 or 48 hrs (bta-miR-193a-3p, 30c and 30b-5p) or up regulation (bta-miR-365-3p) by 48 hrs in the presence of *S. aureus*. Both pathogens presented significant up-regulation of bta-miR-21-3p at 12 hr (1.85 and 1.79 fold change in *E. coli* and *S. aureus*, respectively) and 24 hr (1.68 and 1.55 fold change in *E. coli* and *S. aureus*, respectively) post challenge. In contrast, the expression of bta-miR-21-5p, the other arm of bta-mir-21 and the top expressed miRNA in this study (Table 1) remained constant at these time periods (1.05 and 1.03 fold change in *E. coli* at 12 and 24 hrs respectively, and 1.13 and 1.06 fold change in *S. aureus* at 12 and 24 hrs respectively). The most regulated miRNA after infection was bta-miR-193a-3p (-2.67 fold change in *E. coli* at 6 hrs and -4.93 fold change in *S. aureus* at 48 hrs). It should be noted that in most cases, different miRNAs were differentially regulated at different time points indicating a rapid temporal change in the response pattern of miRNAs to the presence of bacteria. Furthermore, the differential expression pattern of five miRNAs (bta-miR184, miR-24-3p, miR-148, miR-486 and bta-let-7a-5p) were unique to *E. coli* while four (bta-miR-2339, miR-499, miR-23a and miR-99b) were unique to *S. aureus*. The expression of bta-miR-21-3p, miR-365-3p, miR-193a-3p, miR-423-5p and miR-486 in challenged cells was further confirmed by qPCR. The expression trends of these five miRNAs were in most cases similar to results obtained by deep-sequencing (Table 4). For example, bta- miR-193a-3p and miR-365-3p for *E. coli* at 6 h, miR-21-3p and miR-423-5p for *E. coli* at 12 h, miR-423-5p for *E. coli* at 24 h, miR-193a-3p for *E. coli* at 48 h, miR-21-3p for *S. aureus* at 24 h, and miR-193a-3p and miR-365-3p for *S. aureus* at 48 h were similarly differentially expressed (P < 0.05) with both methods. Surprisingly however, was the observed significant differential expression of all five miRNAs in *S. aureus* challenged cells at 6 h with qPCR method as opposed to non-significant regulation with next-generation sequencing. Similarly, different expression patterns of bta-miR-21-3p for *E. coli* at 24 h and *S. aureus* at 12 h, miR-486 for *E. coli* at 48 h and miR-423-5p for *S. aureus* at 12 h between the two methods were also observed.

**Table 3:** **Significantly differentially expressed miRNAs in MAC-T cells between treatments (challenged with *E. coli *** **or *S. aureus *** **bacteria) and controls at same time points**

**Time points**	***E. coli***				***S. aureus***			
	**miRNA ID**	**Fold change**	**log2Fold change**	**P-Value**	**miRNA ID**	**Fold change**	**log2Fold change**	**P-Value**
6 h	bta-miR-193a-3p	0.37	-1.416	4.30E-06	bta-miR-2339	4.56	2.188	0.0427
	bta-miR-365-3p	1.52	0.608	4.47E-03				
	bta-miR-184	1.27	0.343	0.019				
	bta-miR-24-3p	1.33	0.411	0.0229				
	bta-miR-30c	0.75	-0.406	0.024				
	bta-miR-30b-5p	0.71	-0.502	0.048				
12 h	bta-miR-21-3p	1.85	0.886	1.27E-03	bta-miR-21-3p	1.79	0.836	4.20E-03
	bta-miR-148a	1.43	0.516	0.0200	bta-miR-423-5p	0.67	-0.578	0.0168
	bta-miR-423-5p	0.69	-0.545	0.0205	bta-miR-499	0.06	-3.998	0.0300
	bta-miR-92a	1.37	0.454	0.0389	bta-miR-92a	1.36	0.449	0.0428
24 h	bta-miR-423-5p	1.58	0.658	1.21E-03	bta-miR-193a-3p	0.62	-0.700	7.78E-03
	bta-miR-21-3p	1.68	0.751	2.43E-03	bta-miR-23a	1.46	0.550	0.0137
	bta-let-7a-5p	0.69	-0.544	3.43E-03	bta-miR-99b	0.69	-0.540	0.0154
	bta-miR-184	0.76	-0.394	0.0326	bta-miR-21-3p	1.55	0.636	0.0191
	bta-miR-un5	0.52	-0.940	0.0444	bta-miR-un5	0.47	-1.077	0.0238
48 h	bta-miR-486	1.94	0.955	6.46E-04	bta-miR-193a-3p	0.20	-2.302	4.49E-08
	bta-miR-193a-3p	0.64	-0.650	0.0183	bta-miR-365-3p	2.49	1.315	6.87E-04
					bta-miR-30c	0.55	-0.871	0.0191
					bta-miR-30b-5p	0.53	-0.904	0.0241

*Fold change values are as compared to controls at same time points.

**Table 4:** **Results of qPCR validation of the expression of five miRNAs in MAC-T cells between treatments (challenged with *E. coli *** **or *S. aureus *** **bacteria) and controls at same time points**

**Time points**	**miRNA ID**	***E. coli***			***S. aureus***		
		Fold change*	**SE**	**P-Value**^**#**^	**Fold change**	**SE**	**P-Value**
6h	bta-miR-193a-3p	0.342	0.039	**0.021**	0.630	0.054	**0.048**
	bta-miR-21-3p	1.161	0.206	0.508	3.478	0.365	**0.006**
	bta-miR-365-3p	1.607	0.037	**0.014**	0.612	0.040	**0.042**
	bta-miR-423-5p	1.682	0.218	**0.042**	2.018	0.096	**0.008**
	bta-miR-486	2.109	0.304	**0.036**	1.616	0.046	**0.008**
12h	bta-miR-193a-3p	0.992	0.023	0.911	0.601	0.085	0**.010**
	bta-miR-21-3p	2.154	0.276	**0.017**	1.062	0.272	0.819
	bta-miR-365-3p	0.930	0.115	0.568	0.994	0.106	0.962
	bta-miR-423-5p	0.629	0.046	**0.004**	0.812	0.106	0.111
	bta-miR-486	1.614	0.168	**0.032**	1.630	0.342	0.142
24h	bta-miR-193a-3p	1.206	0.052	0.017	1.327	0.171	0.140
	bta-miR-21-3p	1.942	0.410	0.092	1.222	0.066	**0.040**
	bta-miR-365-3p	1.051	0.358	0.879	0.890	0.096	0.478
	bta-miR-423-5p	1.838	0.252	**0.046**	1.304	0.297	0.336
	bta-miR-486	1.099	0.030	0.545	0.867	0.119	0.480
48h	bta-miR-193a-3p	0.353	0.053	**0.020**	0.336	0.026	**0.021**
	bta-miR-21-3p	1.083	0.192	0.679	1.105	0.080	0.385
	bta-miR-365-3p	1.612	0.067	**0.006**	2.809	0.204	**0.008**
	bta-miR-423-5p	1.092	0.017	0.130	2.894	0.258	**0.010**
	bta-miR-486	0.940	0.310	0.835	2.189	0.415	0.071

*Fold change values are as compared to controls at same time points.

^#^Bolded numbers indicate the significance with P < 0.05.

### Enriched KEGG pathways and GO functional categories of predicted gene targets of differentially expressed miRNAs in MAC-T cells challenged with *E. coli* or *S. aureus* bacteria

Target gene prediction of 16 out of 17 miRNAs (target genes for bta-miR-un5 not determined) differentially regulated by the presence of pathogens indicates that about 5169 genes may be regulated by these miRNAs (Additional file 7: Table S4). Gene ontology (GO) functional annotation showed that the target genes of differentially regulated miRNAs were significantly (P < 0.05) enriched in different functional groups, namely; cellular process, developmental process, localization, biological regulation, cellular component organization, cell death, multicellular organismal process, metabolic process, establishment of localization and cell growth (Table 5). The highest involvement of target genes was of 14 miRNAs in the cellular and developmental process followed by target genes of 11 miRNAs in localization and of 10 miRNAs in biological regulation. Interestingly, several KEGG pathways were significantly (P < 0.05) enriched by target genes of 14 of the 17 differentially expressed miRNAs (Table 6 and Additional file 8: Table S5). Notably, pathways of the immune system, signal transduction, cellular processes, nervous system, development and pathways in human diseases, especially cancer were significantly enriched by target genes of at least 4 miRNAs (Table 6).

**Table 5:** **Significantly enriched GO (gene ontology) functional annotation of predicted target genes of differentially expressed miRNAs during *E. coli or S. aureus *** **challenge of MAC-T cells**

**GO functional annotation**	**miRNA**	**# genes**	*P-value (**Benjamini P-Value)	**miRNA**	**# genes**	**P-value (Benjamini P-Value)**
Biological adhesion	Bta-miR-23a	58	0.038 (0.11)	-	-	-
Biological regulation	Bta-miR193a-3p	159	0.002 (0.039)	Bta-miR-24-3p	287	0.010 (0.070)
	Bta-miR365-3p	142	5.5E-5 (3.9E-4)	Bta-miR-148a	356	1.7E-8 (1.3E-7)
	Bta-miR-30b-5p/30c	642	4.4E-6 (3.2E-5)	Bta-miR-23a	565	4.0E-9 (4.2E-8)
	Bta-miR-499	135	0.045 (0.11)	Bta-miR-92a	441	7.2E-10 (1.6E-8)
	Bta-miR-99b	31	0.007 (0.060)	-	-	-
Cellular component organization	Bta-miR-30b-5p/30c	235	2.4E-4 (0.001)	Bta-miR-2339	46	0.002 (0.016)
	Bta-let-7a-5p	182	0.040 (0.120)	Bta-miR-23a	192	0.004 (0.016)
	Bta-miR-499	60	0.001(0.008)	Bta-miR-92a	144	0.016 (0.049)
	Bta-miR-148a	127	9.4E-4 (0.005)	Bta-miR-184	22	0.006 (0.080)
Cellular process	Bta-miR193a-3p	207	0.009 (0.096)	Bta-miR-24-3p	411	1.1E-6 (2.3E-5)
	Bta-miR423-5p	337	0.034 (0.160)	Bta-miR-148a	468	2.1E-9 (4.7E-8)
	Bta-miR365-3p	180	5.7E-4 (0.003)	Bta-miR-2339	143	0.002 (0.020)
	Bta-miR-30b-5p/30c	889	1.1E-10 (2.5E-9)	Bta-miR-23a	766	1.1E-13 (2.4E-12)
	Bta-let-7a-5p	732	1.3E-5 (2.5E-4)	Bta-miR-486-5p	140	0.007 (0.067)
	Bta-miR-499	198	8.2E-6 (1.6E-4)	Bta-miR-92a	567	2.0E-7 (1.4E-6)
	Bta-miR-99b	37	0.036 (0.100)	-	-	-
Death	Bta-miR193a-3p	21	0.038 (0.190)	Bta-miR-24-3p	35	0.046 (0.220)
	Bta-miR-30b-5p/30c	70	0.031 (0.083)	Bta-miR-21-3p	7	0.044 (0.930)
	Bta-let-7a-5p	61	0.026 (0.087)	Bta-miR-92a	47	0.037 (0.084)
	Bta-miR-499	21	0.014 (0.045)	-	-	-
Developmental process	Bta-miR193a-3p	71	0.028 (0.190)	Bta-miR-24-3p	151	1.0E-5 (1.0E-4)
	Bta-miR423-5p	114	0.024 (0.150)	Bta-miR-148a	182	3.7E-9 (4.1E-8)
	Bta-miR365-3p	80	5.8E-7 (1.3E-5)	Bta-miR-2339	57	7.1E-4 (0.015)
	Bta-miR-30b-5p/30c	312	9.1E-8 (1.0E-6)	Bta-miR-23a	272	2.2E-8 (1.5E-7)
	Bta-let-7a-5p	241	0.001 (0.008)	Bta-miR-486-5p	56	0.001 (0.022)
	Bta-miR-499	74	4.1E-4 (0.004)	Bta-miR-92a	212	7.8E-8 (8.5E-7)
	Bta-miR-99b	19	0.002 (0.030)	-	-	-
Establishment of localization	Bta-miR423-5p	110	3.4E-4 (0.007)	Bta-let-7a-5p	205	0.004 (0.011)
	Bta-miR-30b-5p/30c	243	9.9E-4 (0.004)	Bta-miR-92a	159	0.003 (0.009)
Growth	Bta-miR-99b	4	0.016 (0.088)	Bta-miR-23a	20	0.026 (0.088)
Localization	Bta-miR423-5p	119	8.8E-4 (0.009)	Bta-miR-148a	147	0.001 (0.005)
	Bta-miR-30b-5p/30c	280	7.0E-5 (3.9E-4)	Bta-miR-2339	49	0.015 (0.078)
	Bta-let-7a-5p	233	7.2E-4 (0.008)	Bta-miR-92a	182	4.8E-4 (0.002)
	Bta-miR-499	68	0.002 (0.008)	-	-	-
Locomotion	Bta-miR193a-3p	14	0.049 (0.200)	Bta-miR-92a	31	0.030 (0.080)
	Bta-miR-499	17	0.002 (0.009)	-	-	-
Metabolic process	Bta-miR365-3p	139	9.6E-4 (0.004)	Bta-miR-148a	334	0.001 (0.006)
	Bta-let-7a-5p	538	0.001 (0.008)	Bta-miR-23a	559	1.8E-6 (9.7E-6)
	Bta-miR-99b	30	0.025 (0.081)	Bta-miR-92a	395	0.039 (0.084)
Multicellular organismal process	Bta-miR365-3p	94	2.3E-5 (2.4E-4)	Bta-miR-24-3p	167	0.050 (0.190)
	Bta-miR-30b-5p/30c	372	0.001 (0.004)	Bta-miR-148a	196	0.005 (0.015)
	Bta-miR-499	84	0.027 (0.077)	Bta-miR-92a	258	2.2E-5 (1.2-E4)
	Bta-miR-99b	20	0.023 (0.095)	-	-	-

*P-Value: The significance of gene-term enrichment with a modified Fisher’s Exact Test.

**Benjamini P-Value: Globally corrects enrichment p-values in order to control family-wide false discovery rate.

**Table 6:** KEGG (Kyoto encyclopedia of genes and genomes) pathways significantly enriched by target genes of 14 out of 17* differentially expressed miRNAs during *E. coli * **or *S. aureus *** **challenge of MAC-T cells**

**KEGG Pathways**	**miR-365-3p**	**miR-30b-5p/30c**	**Let-7a-5p**	**miR-23a**	**miR-148a**	**miR-24-3p**	**miR-92a**	**miR-193a-3p**	**miR-423-5p**	**miR-99b**	**miR-499**	**miR-486**	**miR-2339**
**Immune system**													
B cell receptor signaling pathway	X	X											
T-cell receptor signaling		X	X										
Fc gamma R-mediated, phagocytosis/chemokine signaling	X			X									
**Signal transduction**													
MAPK signaling pathway	X	X		X		X	X	X	X				
ErbB signaling pathway		X	X		X	X							
Wnt signaling pathway	X	X		X	X	X		X					
TGF-beta signaling pathway			X	X	X	X							
Calcium signaling pathway						X	X		X				
Phosphatidylinositol pathway				X			X						
ECM-receptor interaction			X		X								
**Cellular processes (transport and catabolism, cell motility, cell communication)**													
Endocytosis		X		X		X							
Lysosome/apoptosis	X		X										
Regulation of actin cytoskeleton	X	X		X	X	X	X				X		
Cell cycle	X		X		X		X						
P53 signaling pathway			X	X	X							X	
Adherens/tight junction				X									X
Focal adhesion		X	X	X	X	X							
**Endocrine system**													
Insulin signaling pathway			X						X				
Adipocytokine signaling pathway			X		X								
GnRH signaling pathway			X					X	X				
Melanogenesis	X					X				X			
**Nervous system**													
Long term potentiation	X	X					X			X			
Neurotrophin signaling pathway	X	X	X	X		X		X					
**Development:** Axon guidance		X	X	X	X	X			X		X	X	X
**Metabolism:** Glycerophospholipid metabolism/glycosphingolipid biosynthesis				X					X				
**Human diseases**													
Pathways in cancer		X	X	X	X			X	X	X	X	X	
Colorectal cancer	X	X	X	X		X		X		X	X		
Pancreatic cancer	X		X	X	X								
Bladder cancer			X										
Prostate cancer	X	X	X	X	X							X	
Small cell lung cancer	X		X									X	
Non-small cell lung cancer	X	X	X		X								
Thyroid cancer			X										
Renal cell carcinoma	X			X	X			X					
Endometrical cancer	X		X										
Glioma	X	X	X		X							X	
Melanoma	X		X		X							X	
Type II diabetes mellitus		X	X										
Chronic myeloid leukemia	X		X	X	X								
Acute myeloid leukemia					X			X					

*Results for miR-21-3p were ignored because only few target genes out of 78 were non-significantly enriched in two pathways (Neurotophin signaling pathway- 3 genes and Ubiquitin mediated pathway- 3 genes). Similarly, only 3 genes each out of 88 target genes for miR-184 were enriched (P > 0.05) in two pathways, Chronic myeloid leukemia and Prostate cancer and were ignored. The target genes for miR-un5, a newly identified miRNA in this study were not determined.

## Discussion

Next-generation sequencing technologies have not only enabled the detection of more lowly expressed miRNAs and novel miRNA discovery, but also accurate profiling of the expression of miRNAs at a high-throughput level. Using deep-sequencing, we have identified a number of novel bovine miRNAs and revealed a differential regulation of miRNAs in MAC-T cells challenged with *E. coli* or *S. aureus* bacteria, thus suggesting the regulation of miRNAs in host response to these mastitis pathogens.

The number of miRNAs deposited in miRBase has increased exponentially in recent years and high throughput sequencing has contributed to almost all sequences submitted to miRBase after 2007 [30]. However, high throughput sequencing generates millions of reads per sample and requires powerful and accurate computational methods to mine known and novel miRNAs. Several computational software packages such as miRDeep [28], MIReNA [31], miRanalyzer [32], and miRTRAP [33] have been developed to identify miRNAs from high-throughput data. Our study used a combination of miRDeep2 and Blastn and identified 113 novel miRNAs in MAC-T cells. It is notable that computational based approaches may over-predict novel miRNAs and our data remain to be validated by further studies.

It is necessary to give a consistent and unique gene name for each novel miRNA. The nomenclature of miRNA has been well described previously [30,34,35]. However, the annotation of miRNAs still presents misperceptions in some instances. Some recently identified miRNAs and their precursors are reversely complementary to previously known miRNAs and precursors. For example, hsa-mir-103a and hsa-mir-103b (miRBase Release 19) are paired (reversely complementary) but are usually thought to be distinguished by 1 or 2 nucleotide differences in their nucleotide sequences. Here we added a symbol “`” after miRNA to represent the antisense of a known miRNA precursor (Table 2), *i.e.* hsa-mir-103 and mir-103′instead of mir-103a, b, respectively. In addition and to the best of our knowledge, there is no established criterion that defines conserved miRNAs. We suggest that a novel miRNA is homologous or orthologous if: 1) it precursor aligns to a known miRNA precursor with > 70% identity and > 75% sequence coverage; or 2) it has > 90% identity and > 90% length coverage with a known miRNA as well as same arm location on the precursor. Previously, only sequence similarity was taken into account for the annotation of conserved miRNAs and disparities have been known to occur. For example, bta-miR-193a [36] (miRBase Release 19), generated from bta-mir-193a-2, is reversely complementary to bta-miR-193a-3p from 1 to 19 nucleotides but not correctly named. Using this criterion, one miRNA candidate identified in this study (un219, Additional file 3: Table S3) was aligned to bta-miR-222. Un219 is at the same arm as bta-miR-222 on the precursor and therefore named as bta-miR-222b (Table 2, Additional file 3: Table S3).

Only a few miRNAs (Table 1) were highly expressed, accounting for 78.55% of all known bovine miRNAs detected. This observation is consistent with a recent study that detected similar miRNAs expression levels in bovine primary mammary epithelial cells infected with *Streptococcus uberis* and also, seven of the top expressed miRNAs in that study [25] are the same as in this study. Most of the detected top expressed miRNAs are conserved in human, mouse, and bovine, and belong to several miRNA families, vis, miR-31, miR-26a, miR-27a-3p/27b, let-7a-5p/7f/7i, miR-21-5p, miR-22-3p, miR-184, miR-186, miR-191, miR-205 and miR221/222. In particular, several of these highly expressed miRNAs are known to play roles in growth and immunity. The top expressed miRNA, bta-miR-21-5p in this study, was also the most highly expressed miRNA in human blood monocytes challenged with *Mycobacterium leprae* and also found to negatively regulate the vitamin D-dependent antimicrobial pathway in leprosy [37]. miR-21-5p has been shown to be induced by LPS and to negatively regulate TLR4 by targeting the programmed cell death protein 4 (PDCD4) and to promote the expression of interleukin 10 (IL-10) [38]. Further evidence of the immune capacity of miR-21 was from the study of Narducci et al. [39] who provided *in vitro* evidence for involvement of miR-21, miR-214 and miR-486 in cell survival in Sézary syndrome in humans. It has been recently shown that the level of immune and development related miRNAs, including miR-27b, were significantly higher in colostrum than in mature milk [40]. Up-regulation of miR-27b by LPS was found to destabilize proliferator-activated receptor γ1 (PPARγ1) mRNA which is often associated with chronic inflammatory diseases [41]. In addition, miR-27b was reported to target KSRP and increase iNOS mRNA stability for host’s defense against cryptosporidial infection [17], further supporting a role for this miRNA in immunity. Therefore, the highly expressed miR-21-5p and miR-27b in mammary epithelial cells in this study might be associated with a function in immunity.

Five miRNAs were found to be significantly regulated during the 48 hr cell incubation without pathogens. It is not surprising owing to their involvement in almost all biological processes as demonstrated by GO functional annotation of the target genes of three (bta-miR-193a-3p, miR-423-5p and miR-30b-5p) of these miRNAs. GO functional annotation of target genes of bta-miR-193a-3p and miR-30b-5p showed enriched genes related to cell growth and death, *e.g.* growth arrest specific gene 1 (GAS1), myeloid cell factor 1 (MCL1, also BCL2 related), BCL2-like 11 (apoptosis facilitator) and programmed cell death 10 (PDCD10). GAS1 has been shown to be involved in growth suppression through blocking of entry to S-phase and also prevents cycling of normal and transformed cells [42,43]. Also, involvement of MCL1 and PDCD10 in the regulation of apoptosis and cell survival has been demonstrated [44-46]. It is well known that cells enter the stationary phase after reaching confluence. Additionally, we used fetal bovine serum (FBS) free media, known to inhibit proliferation, during the 48 hr period of cell incubation. The up-regulation of miR-193a-3p and miR-30b-5p may play regulatory roles in cell death which need to be further confirmed in the context of mastitis.

Out of the 231 known and 113 novel miRNAs identified in this study, only 17 showed differential expression after challenge of MAC-T cells with *E. coli* or *S. aureus* bacteria, which may look small considering the large number of identified miRNAs. It is probable that observed miRNA expression profiles in response to heat inactivation of bacteria in this study may be different than expression elicited by live bacterial cells. In a recent study [47], a substantially different miRNA expression profile emerged from killed virulent bacilli as compared to live active bacteria thus suggesting an active influence of living bacteria on cellular miRNA metabolism. However, our results on response to heat inactivated *S. aureus* are in line with literature information whereby 21 miRNAs were shown to be differentially expressed after challenge of bovine primary mammary epithelia cells with live *S. uberus* (Gram-positive) bacteria [25]. Regardless, further studies on the miRNA expression patterns during infection with live and active bacteria is necessary to verify the miRNA expression variation identified between these two species. In addition, there were discrepancies in expression pattern of some low abundant miRNAs during the infection detected by sequencing as compared to RT-PCR. This difference may be due to the pooling approach used for library preparation and subsequent sequencing, as well as the relative low sequence reads which may limit the proper detection of the low abundant miRNAs by miRNA-Seq. Furthermore, the miRNA signature in different organs, tissues or cell types is different and also regulated differently. miRNA expression profile has been shown to vary between mature milk and colostrum [48], between lactating and non-lactating mammary glands [49] and, between organs (lung, brain, liver and spleen) and whole blood [50]. During the onset of mastitis and subsequent progression, the whole system reacts by recruiting immune cells into the mammary gland to help combat infection. These immune cells and other factors, including resident factors and cells in the mammary gland work in concert to ward off infection. It should be noted here that, the mammary epithelial cells used in this study only constitutes a small portion of that army of cell types that secrete defence factors against invading pathogens. Thus, the present findings portray only the role of epithelial cells derived miRNAs in the fight against mastitis pathogens, which may be different from the holistic picture that can be obtained after examination of all players. In profiling the transcriptome of *S. uberis* induced mastitis, fundamental differences were revealed between immune gene expression in the mammary gland and in a primary cell culture model thus demonstrating the complexity of the bovine mammary gland immune response to an infecting pathogen, and also indicating that a coordinated response exists between resident, recruited, and inducible immune factors [51]. Furthermore, transcriptomics comparisons between MAC-T cells and mammary tissue during late pregnancy and peak lactation showed a larger overall similarity in gene expression between MAC-T cells and lactating than non-lactating mammary tissue [52]. Despite these demonstrated differences [51,52] and or similarities [52], our results showed similarity between highly expressed miRNA profiles with primary bovine mammary epithelial cells challenged with Gram positive bacteria [25] and non-challenged mammary gland tissues (data not shown) thus indicating that the MAC-T cell is still a valuable tool to study mammary gland biology. Importantly, the findings from our study show the positive involvement of mammary epithelia cells in contributing to the immune response to infecting pathogens.

Furthermore, our study has demonstrated that *E. coli* initiated a stronger response at the early hours of infection as demonstrated by the differential response of six miRNAs within the first six hours of cell challenge with *E. coli* bacteria as opposed to only one differentially expressed miRNA in the presence of *S. aureus*. By 48 hrs, two miRNAs were differentially regulated in *E. coli* challenged cells while in *S. aureus* cells, more miRNAs were implicated with time. Furthermore, three of the miRNAs that responded significantly to the presence of *E. coli* at 6 hrs post infection only showed a differential response in *S. aureus* cells by 24 or 48 hrs. Interestingly, our study shows that a different set of five miRNAs (miR-184, miR-24-3p, miR-148, miR-486 and let-7a-5p) were unique to *E. coli* bacteria while another set of four (miR-2339, miR-499, miR-23a and miR-99b) were unique to *S. aureus* bacteria. Our data therefore demonstrate a differential response pattern of MAC-T cells to Gram-negative *E. coli*, as compared to Gram-positive *S. aureus*. Mastitis caused by pathogenic ligands from Gram-positive or Gram-negative bacteria usually elicits different response patterns from the host thus leading to different degrees of mastitis. Our data supports the intense immune reaction usually caused by *E. coli* mastitis, which often leads to acute mastitis. On the contrary, the slower initial response of miRNAs to *S. aureus* bacteria may support the slow progression of mastitis caused by this type of bacteria. Differential response patterns of miRNAs to Gram-positive and Gram-negative bacteria have been demonstrated previously [22,49]. Furthermore, our data is supported by previous studies that have demonstrated pathogen-dependent differences of host (mammary gland) responses to other immune factors (genes and proteins) [4,7-10].

It is notable that predicted gene targets of most of the differentially expressed miRNAs in response to pathogens in this study are significantly enriched for GO functionally annotated roles like developmental process, cellular process, localization, biological regulation, cellular component organization, multicellular organismal process, cell death, metabolic process, establishment of localization and growth, thus supporting the involvement of these miRNAs in numerous developmental and physiological processes, including disease development. This also goes a long way to portray the significant roles of miRNAs in supporting the mammary gland in its growth and productive capacities as well as defense capabilities. Interestingly, pathway analysis of miRNA predicted gene targets, showed significant enrichments in KEGG pathways already associated with host response to different diseases. Notably, many of the KEGG pathways significantly enriched by differentially expressed miRNA target genes in this study have been previously associated to mammary gland responses to bacterial pathogens in bovine [6,23,25]. For example, gene targets of five differentially expressed miRNAs (miR-365-3p, miR-30b-5p, miR-30c, let-7a-5p and miR-23a) were enriched for pathways in immune system (B-cell receptor signaling pathway, chemokine signaling, T-cell receptor signaling and Fc gamma R-mediated phagocytosis). These miRNAs are amongst several miRNAs with demonstrated roles in immunity. A recent study showed that the expression levels of three miRNAs including miR-23a were significantly higher in breast cancer with lymph node metastasis, compared with that from patients without lymph node metastasis or normal tissue and also that the expression of the miR-23a/24-2/27a cluster promoted mammary carcinoma cell migration, invasion, and hepatic metastasis, through targeting Sprouty2 (SPRY2) and consequent activation of p44/42 MAPK (mitogen-activated protein kinase) [53]. The MAPK pathway was highly activated in mammary cells and tissues challenged with *S. uberis*[23,25]. In addition, the role of Let-7 miRNA family in immunity is well established. The let-7 miRNA family was identified as the common denominator of Salmonella-regulated miRNAs in macrophages and epithelial cells, thus suggesting that repression of let-7 relieves cytokine IL-6 and IL-10 mRNAs from negative post-transcriptional control, thus establishing a paradigm of miRNA-mediated feed-forward activation of inflammatory factors when mammalian cells are targeted by bacterial pathogens [54]. Let-7b/d/e miRNAs, were recently shown to be differentially regulated in infected (*S. uberis*) bovine mammary cells [25].

## Conclusion

Using deep sequencing, we characterized the miRNome of bovine mammary epithelia cells (MAC-T cells) challenged with heat inactivated *E. coli* or *S. aureus* bacteria and detected 231 known bovine miRNAs and 113 novel miRNAs, and also a pathogen dependent differential regulation of miRNAs. In particular, E*. coli* elicited an earlier differential regulation of miRNAs as opposed to a delayed regulation by *S. aureus*. Highly expressed and differentially regulated miRNAs have demonstrated roles in diverse biological processes as well as in immunity. GO and KEGG pathway analysis showed significant enrichments of predicted target genes of differentially regulated miRNAs in different functional groups (e.g. cellular process, developmental process, biological regulation, cell death, etc.) and KEGG pathways of the immune system, signal transduction, cellular process, nervous system, development and pathways in cancer and other human diseases. Importantly, our study provides further confirmation of the involvement of mammary epithelia cells in contributing to the immune response to infecting pathogens and a potential role for miRNAs as biomarkers in early diagnosis of mastitis and in development of control measures.

## Methods

### Cell culture and bacteria challenge

Cell culture and bacteria challenge were carried out as previously described with some modifications [4]. Briefly, MAC-T cells (a bovine mammary epithelial cell line) were seeded at a concentration of 1.5 × 10^5^ cells in a 6 well cell culture plate (BD Biosciences, Mississauga, Ontario, Canada) and grown in a growth medium for 24 hours at 37°C in 5% CO_2_ humidified incubator. The growth medium contained DMEM and RPMI 1640 (Wisent, St-Bruno, Quebec, Canada) at a concentration of 1:1, 10% fetal bovine serum (FBS) (Wisent), 10 μL/mL ITS (insulin-transferrin-selenium solution) (Wisent), and 1% antibiotic antimycotic solution (100×) (Wisent). At 85% confluence, the growth medium was changed for infection medium (same as the growth medium without FBS) and cells were allowed to grow for another 24 hr period before infection with bacterial pathogens.

*Escherichia coli* (*E. coli*) strain P4 and *Staphylococcus aureus* (*S. aureus*) strain Smith CP were the infection agents. Bacteria were initially grown overnight on Luria Bertani (LB) agar (*E. coli*) or on tryptic soy agar (TSA) (*S. aureus*) aerobically in a humidified incubator at 37°C. A single colony of *S. aureus* was transferred to a 50 mL conical tube containing 20 ml of tryptic soy broth (TSB) and incubated at 37°C in an open air shaker at 225 RPM. Similarly, a single colony of *E. coli* was grown the same way in LB broth. The bacteria were grown until an OD_600nm_ of 0.6 was reached and then plated in triplicates on their respective media to confirm the number of bacteria per mL. Growth of bacteria and subsequent manipulations were carried out in the biosafety containment level II laboratory of the Dairy and Swine Research and Development Centre of Agriculture and Agri-Food Canada, Sherbrooke, following institutional safety procedures.

Prior to infection, cells in 6 wells were individually trypsinized and counted using the Countess^®^ Automated Cell Counter (Life Technologies, Burlington, Ontario, Canada). The two bacterial strains were then diluted to achieve a concentration enabling a ratio of 10 bacteria to 1 MAC-T cell in the infection medium and then heat inactivated at 63°C for 30 minutes to prevent overgrowth during the period of cell challenge. After refreshing infection medium, each triplicate cell well representing different time points (6, 12, 24 and 48 hr) and each bacterial strain was challenged with the infection medium containing bacteria to achieve an infection rate of 1:10. Non-challenged triplicates (control) for each time point (0, 6, 12, 24 and 48 hr) were also included. Uninfected cells were treated with the same volume of heat inactivated infection media without bacteria. After 0, 6, 12, 24 and 48 hr, the media were removed, cells washed once in Hank’s balanced salt solution 1X (HBSS 1X; Wisent) without trypsinisation, harvested in 1 mL of lysis/binding buffer (mirVana miRNA Isolation Kit, Ambion Inc., Austin, TX, USA) and stored at -80°C until RNA extraction.

### Total RNA isolation and library construction

Total RNA was extracted using the mirVana miRNA Isolation Kit (Ambion^®^, life technologies, USA) following manufacturer’s protocol. The concentration and purity of the isolated RNA was checked by NanoDrop^®^ ND-1000 Spectrophotometer (NanoDrop Technologies Inc., Wilmington, DE, USA). The quality of RNA was further assessed with the RNA 6000 Nano Labchip Kit using the Agilent 2100 Bioanalyzer (Agilent Technologies, CA, USA). All the 39 RNA samples had a RNA integrity number (RIN) of 9.7-10 and were stored at -80°C until further analysis.

Equal amounts (330 ng) of total RNA from triplicate samples at the same time point were pooled to construct small RNA library using the TruSeq Small RNA Sample Preparation kit (Illumina, San Diego, CA, USA) according to the manufacturer’s instruction. PCR amplification was performed for 13 cycles. In total, 13 small RNA libraries were constructed and pooled in equal amounts for gel purification and sequencing. Sequencing was carried out on the HiScan SQ system (Illumina) using the TruSeq™ SBS kit v3 (50 cycles, Illumina). Real-time analysis and base calling was done using the HiSeq Control Software Version 1.4.8 (Illumina).

### Small RNA sequence analysis

Low-quality reads were removed from the raw reads using CASAVA 1.8 based on chastity. After trimming the 3′ adaptor sequence by a perl script (“clip_adapters.pl”) provided by miRDeep2 software (version 2.0.0.5), small RNA reads with 18–30 nts in length from all libraries were extracted and pooled to identify known and novel miRNAs using miRDeep2 with the default parameters [28]. All reads were aligned to the bovine genome (2009, UMD3.1) with Bowtie (allowing 1 mismatch) [55] and reads that mapped to bovine rRNAs, tRNAs, and snoRNAs in the Rfam RNA family database [56] were discarded. The remaining reads were scored by miRDeep2 as known or potential novel bovine miRNAs. Small RNAs that mapped only to genomic repeat loci were removed. The lowest score cut-off of 5 that yielded a signal-to-noise ratio of 16.2 [28] was used for novel miRNA predication and small RNAs with higher scores than threshold were regarded as potential novel miRNAs. Furthermore, small RNAs having lower scores than cut-off but more than 10 reads in total [30] were further analyzed as miRNA candidates. Small RNAs < 20 nucleotides in size were ignored because of higher false-positive discovery rates and the remaining novel miRNAs and miRNAs candidates were further annotated using Blastn [57] (word size = 6). Conserved novel miRNAs were classified as homologous known miRNAs or orthologous known bovine miRNAs and named according to the following guidelines: (1) it matches > 90% identity and > 90% of the length of a known animal miRNA (miRBase Release 19), or (2) it precursor aligns to any precursor of a known miRNA with > 70% identity and > 75% sequence coverage [58]. In addition, a huge bovine specific miRNA family (bta-miR-2284, miRBase Release 19) containing more than 80 members, shows very high similarity in their precursor sequences. Therefore, novel miRNAs were regarded as new members of bta-miR-2284 family if their precursors were clustered by CLUSTAL W [59] with bta-miR-2284 precursors. Non-conserved novel miRNAs were also named and inputted into miRBase, while the remaining miRNA candidates that didn’t follow any of the categories above were not further analyzed.

The expression of miRNAs (known and novel miRNAs) for each library was calculated by miRDeep2 using the Quantifier module [28]. Reads that mapped equally well to the positions of two or more mature miRNAs were divided equally to the read counts of those mature miRNAs. To investigate the regulation of miRNAs in MAC-T cells in response to stimulation by pathogens, miRNA expression in challenged cells at different time points were compared to expression in unchallenged cells (controls) at the same time point using DEseq and raw reads as suggested [29]. DEseq allows a P-value to be determined in the absence of any available biological replicates, by treating the two conditions as replicates, under the assumption that only a small proportion of transcripts are differentially expressed [29]. Similarly, miRNA regulation in control cells over time was determined by comparing expression levels at different time points and 0 hr.

### Target gene prediction and pathway analysis

The putative target genes of differentially expressed miRNAs were predicted using Ingenuity Pathway Analysis (IPA) software (Ingenuity Systems Inc., Redwood City, CA, USA). IPA uses a miRNA target filter that depends on experimentally validated gene-miRNA interactions from TarBase database (http://diana.cslab.ece.ntua.gr/tarbase) and miRecords (http://mirecords.biolead.org/), and the presence of conserved 8mer and 7mer sites on 3 prime untranslated regions (3′UTR) of genes that match the seed region of each miRNA from TargetScan (http://www.targetscan.org). Additionally, manually curated findings within the IPA knowledgebase are also used. Predictions returned by IPA are either experimentally validated interactions, high or moderate interactions between the 8mer/7mer seed regions of miRNA that match sites on 3′UTR of genes. In this study, only genes showing high predictions and experimentally validated interactions were further analysed. Predicted gene targets were imputed into the Database for Annotation, Visualization and Integrated Discovery (DAVID version 6.7, http://david.abcc.ncifcrf.gov/) for gene ontology (GO) functional annotation and pathway analysis.

### Quantitative PCR and data analysis

Five differentially expressed miRNAs (bta-miR-193a-3p, miR-423-5p, miR-21-3p, miR-365-3p and miR-486) were validated by quantitative RT-PCR using TaqMan^®^ miRNA Assays following manufacturer’s recommendations (Applied Biosystems, Foster City, CA, USA). Briefly, cDNA was reversely transcribed from 10 ng of total RNA using 5 × specific miRNA RT primer for each miRNA. PCR products were amplified from cDNA samples using 20 x TaqMan^®^ miRNA Assays. Fluorescence signals were detected on an ABI StepOnePlus Real-time PCR System (Applied Biosystems). Triplicates for each reaction were performed. Bovine miR-192 was used as internal control since it was the best stable miRNA detected by sequencing (C.V =5.14%). The relative expression of target miRNA was calculated following the formula:

ΔCt_target miRNA_ = Ct_target miRNA_–Ct_internal control_. The control group at each time point was selected as a calibrator in each comparison group for the relative quantification. The relative expression of miRNA in a sample normalized to internal control and relative to the calibrator was calculated as follows: Relative expression _target miRNA_ = 2^-ΔΔCt^, where ΔΔCt = ΔCt _target miRNA, sample_ -ΔCt_target miRNA, calibrator_. T-test (SAS version 9.2, 2008) was used to compare the difference between each comparison group. Significant differential expression was declared at P < 0.05.

### Availability of supporting data

All the sequencing data sets supporting the results in this study have been deposited in the publicly available NCBI’s Gene Expression Omnibus Database (http://www.ncbi.nlm.nih.gov/geo/). The data are accessible through GEO Series accession number GSE51979 (http://www.ncbi.nlm.nih.gov/geo/query/acc.cgi?acc=GSE51979).

## Competing interests

The authors declare that they have no competing interests.

## Authors’ contributions

LLG, WJ, EMIA and XZ designed the experiments and wrote the paper. WJ constructed the libraries and carried out the qRT-PCR. GL did the data analysis and contributed to manuscript writing. EMIA performed target gene predictions and pathway analysis. FB performed the cell culture experiments. All authors read and approved the final manuscript.

## Supplementary Material

Additional file 1: Table S1 Known miRNAs identified in MAC-T cells challenged with or without *E. coli* or *S. aureus* bacteria by miRDeep2. This table contains three sheets. Click here for file

Additional file 2: Table S2 Novel miRNAs predicted by miRDeep2 in MAC-T cells challenged with or without *E. coli* or *S. aureus* bacteria. Click here for file

Additional file 3: Table S3 miRNA candidates detected in MAC-T cells challenged with or without *E. coli* or *S. aureus* bacteria by miRDeep2. Click here for file

Additional file 4: Figure S1 New members of bta-mir-2284 family identified from precursors of miRNA candidates. The precursors of miRNA candidates were clustered by CLUSTAL W. Click here for file

Additional file 5: Figure S2 Correlations in miRNA expression between libraries. Click here for file

Additional file 6: Figure S3 Confirmation of the expression of bta-miR193a-3p and miR-423-5p in control cells by qRT-PCR. Click here for file

Additional file 7: Table S4 Predicted target genes of differentially expressed miRNAs in MAC-T cells challenged with *E. coli* or *S. aureus* bacteria. This table contains 15 sheets. Click here for file

Additional file 8: Table S5 KEGG pathways and disease terms significantly enriched by target genes of differentially expressed miRNAs during *E. coli* or *S. aureus* challenge of MAC-T cells. Click here for file
