# Supplementary figures and images for: Transcriptome microRNA profiling of bovine mammary epithelial cells challenged with Escherichia coli or Staphylococcus aureus bacteria reveals pathogen directed microRNA expression profiles
Source: BMC Genomics. 2014 Mar 7;15:181. doi: 10.1186/1471-2164-15-181 (PMC4029070; doi:10.1186/1471-2164-15-181)

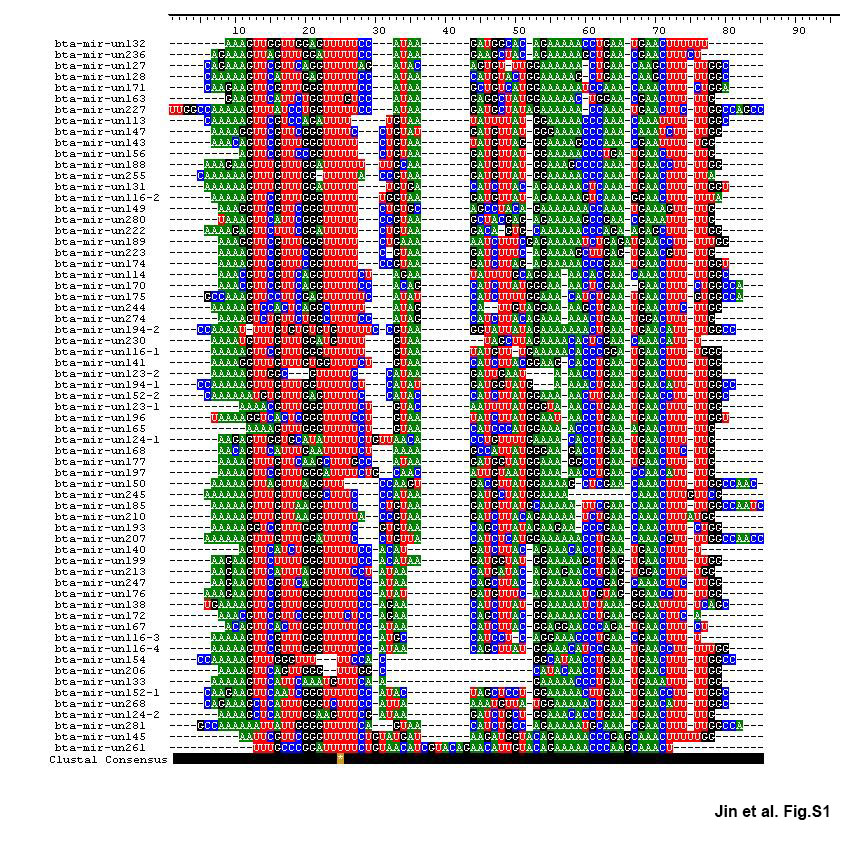

Supplement: Additional file 4: Figure S1 — New members of bta-mir-2284 family identified from precursors of miRNA candidates. The precursors of miRNA candidates were clustered by CLUSTAL W. [file 1471-2164-15-181-S4.JPEG]

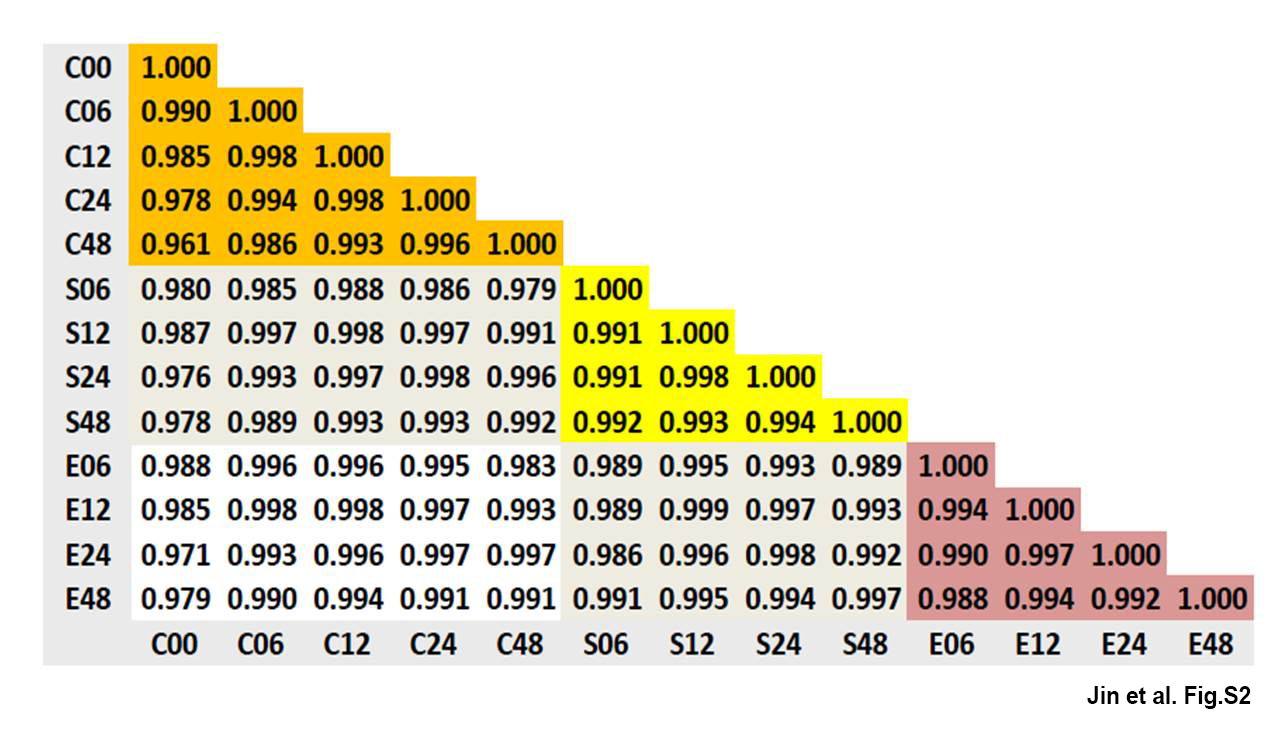

Supplement: Additional file 5: Figure S2 — Correlations in miRNA expression between libraries. [file 1471-2164-15-181-S5.JPEG]

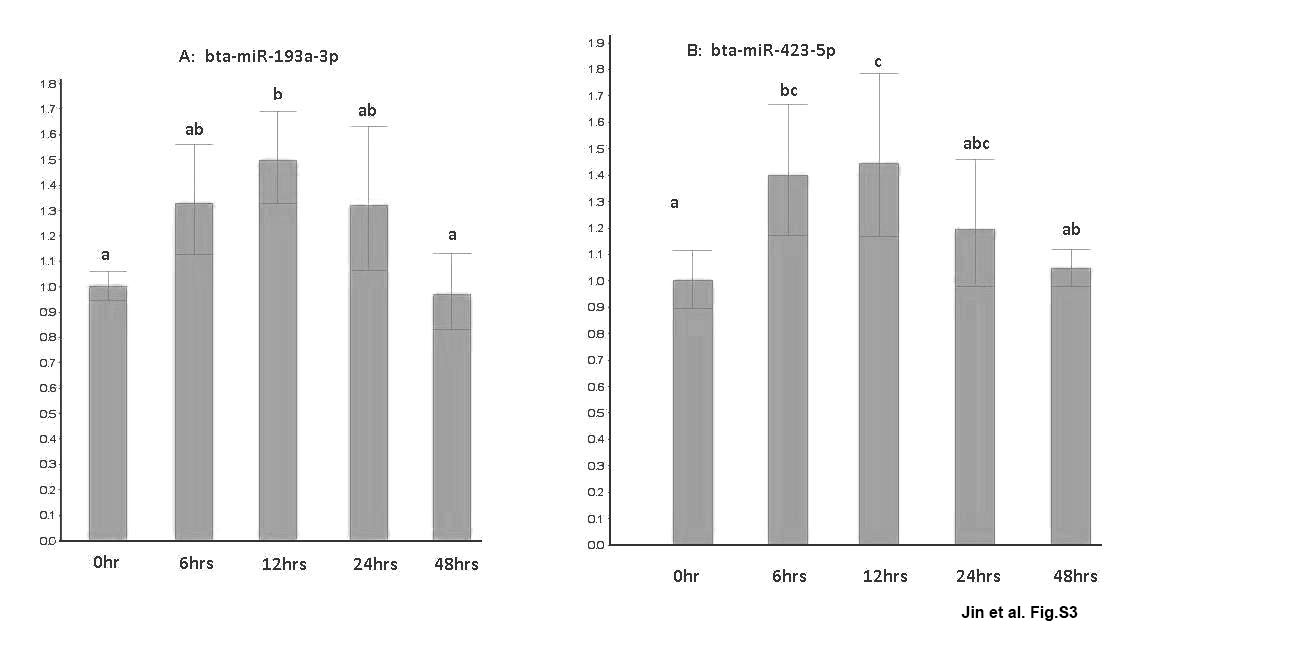

Supplement: Additional file 6: Figure S3 — Confirmation of the expression of bta-miR193a-3p and miR-423-5p in control cells by qRT-PCR. [file 1471-2164-15-181-S6.JPEG]
