# Supplementary material for: Transcriptome microRNA profiling of bovine mammary epithelial cells challenged with Escherichia coli or Staphylococcus aureus bacteria reveals pathogen directed microRNA expression profiles
Source: BMC Genomics. 2014 Mar 7;15:181. doi: 10.1186/1471-2164-15-181 (PMC4029070; doi:10.1186/1471-2164-15-181)
Supplement: Additional file 8: Table S5 — KEGG pathways and disease terms significantly enriched by target genes of differentially expressed miRNAs during E. coli or S. aureus challenge of MAC-T cells. [file 1471-2164-15-181-S8.DOCX]

**Table S5:** Kegg pathways and disease terms significantly enriched by target genes of differentially expressed miRNAs during *E. coli* or *S. aureus* challenge of MAC-T cells.

| miRNA | Kegg pathway | # genes | *P-Value (**Benjamini P-value) | Disease |
| --- | --- | --- | --- | --- |
| Bta-miR-30b-5p  Bta-miR-30c | Axon guidance | 24 | 2.5E-5 (0.004) | Schizophrenia, bipolar disorder, autism, body mass, hearing loss (deafness), Schizoaffective disorder, bipolar disorder, methamphetamine abuse, type 2 diabetes, diurnal preference, hypertension, longevity, weight gain, retinal vascular occlusion, insulin |
|  | Neurotophin signaling pathway | 23 | 4.1E-5 (0.003) |  |
|  | Ubiquitin mediated proteolysis | 22 | 5.1E-4 (0.026) |  |
|  | Long term potentiation | 14 | 7.5E-4(0.028) |  |
|  | Amytrophic lateral sclerosis | 12 | 9.4E-4 (0.028) |  |
|  | B cell receptor signaling pathway | 14 | 0.002 (0.048) |  |
|  | Pathways in cancer (colorectal cancer, non-small cell lung cancer, prostate cancer) | 33 | 0.002 (0.042) |  |
|  | Arrhythmogenic right ventricular cardiomyopathy | 14 | 0.002 (0.041) |  |
|  | Regulation of actin cytoskeleton | 27 | 0.004 (0.070) |  |
|  | Dilated cardiomyopathy | 15 | 0.005 (0.068) |  |
|  | Hypertrophic cardiomyopathy | 14 | 0.006 (0.080) |  |
|  | Aldosterone-regulated sodium reabsorption | 9 | 0.007 (0.084) |  |
|  | Wnt signaling pathway | 20 | 0.009 (0.100) |  |
|  | Glioma | 11 | 0.012 (0.120) |  |
|  | SNARE interactions in vesicular transport | 8 | 0.015 (0.150) |  |
|  | ErbB signaling pathway | 13 | 0.018 (0.160) |  |
|  | Endocytosis | 21 | 0.033 (0.260) |  |
|  | MAPK signaling pathway | 28 | 0.034 (0.240) |  |
|  | T-cell receptor signaling pathway | 14 | 0.039 (0.250) |  |
|  | Focal adhesion | 22 | 0.043 (0.260) |  |
|  | Type II diabetes mellitus | 8 | 0.044 (0.260) |  |
| Bta-let-7a-5p | MAPK signaling pathway | 42 | 6.7E-8 (1.1E-5) | Breast cancer , melanoma, bone mass, esophageal cancer, colon cancer, colorectal cancer, heart failure, prostate cancer, blood pressure, arterial hypertension, ovarian cancer, bone mineral density, bladder cancer, heart rate, resting, body mass (obesity, localised), cardiomyopathy (heart failure), periodontitis, metabolism disorders, longevity, chromosomal damage, multiple sclerosis, osteoarthritis, stomach cancer, chronic obstructive pulmonary disease, bone density (fracture, vertebral), type 2 diabetes (insulin), lymphoma, preterm delivery, Alzheimer’s disease, liver transplant, hypertension, heart transplant |
|  | Chronic myeloid leukemia | 18 | 3.1E-6 (2.5E-4) |  |
|  | Pathways in cancer (pancreatic cancer, colorectal cancer, bladder cancer, small cell lung cancer, prostate cancer, non-small cell lung cancer, thyroid cancer, endometrical cancer) | 41 | 3.7E-5 (0.002) |  |
|  | Melanoma | 15 | 1.2E-4 (0.005) |  |
|  | Glioma | 14 | 1.3E-4 (0.004) |  |
|  | P53 signaling pathway | 14 | 2.9E-4 (0.005) |  |
|  | Neurotrophin signaling pathway | 17 | 0.005 (0.079) |  |
|  | Hypertrophic cardiomyopathy | 13 | 0.007 (0.092) |  |
|  | Adipocytokine signaling pathway | 11 | 0.009 (0.110) |  |
|  | Focal adhesion | 22 | 0.016 (0.160) |  |
|  | Axon guidance | 16 | 0.016 (0.160) |  |
|  | ECM-receptor interaction | 12 | 0.017 (0.170) |  |
|  | GnRH signaling pathway | 13 | 0.017 (0.170) |  |
|  | TGF-beta signaling pathway | 12 | 0.021 (0.160) |  |
|  | Heparan sulfate biosynthesis | 6 | 0.022 (0.160) |  |
|  | Insulin signaling pathway | 16 | 0.024 (0.160) |  |
|  | Cell cycle | 15 | 0.027 (0.180) |  |
|  | Type II diabetes mellitus | 8 | 0.028 (0.170) |  |
|  | Dilated cardiomyopathy | 12 | 0.031 (0.180) |  |
|  | Lysosome | 14 | 0.034 (0.200) |  |
|  | O-Glycan biosynthesis | 6 | 0.039 (0.390) |  |
|  | T cell receptor signaling pathway | 13 | 0.041 (0.210) |  |
|  | Progesterone-mediated oocyte maturation | 11 | 0.045 (0.220) |  |
|  | Chondroitin sulfate biosynthesis | 5 | 0.048 (0.220) |  |
|  | ErbB signaling pathways | 11 | 0.049 (0.220) |  |
|  |  |  |  |  |
| Bta-miR193a-3p | GnRH signaling pathway | 8 | 0.001 (0.140) | Alzheimer’s disease |
|  | Pathways in cancer (colorectal cancer, renal cell carcinoma) | 14 | 0.004 (0.180) |  |
|  | Neurotrophin signaling pathway | 8 | 0.005 (0.170) |  |
|  | Wnt signaling pathway | 8 | 0.014 ()0.230 |  |
|  | Acute myeloid leukemia | 5 | 0.017 (0.240) |  |
|  | Melanogenesis | 6 | 0.027 (0.310) |  |
|  | MAPK signaling pathway | 10 | 0.038 (0.350) |  |
|  | Oocyte meiosis | 6 | 0.019 (0.340) |  |
| Bta-miR423-5p | MAPK signaling pathway | 18 | 0.004 (0.390) | Thyrotoxic periodic paralysis |
|  | Glycerophospholipid metabolism | 8 | 0.005 (0.280) |  |
|  | Axon guidance | 11 | 0.007 (0.260) |  |
|  | GnRH signaling pathway | 9 | 0.011 (0.310) |  |
|  | Insulin signaling pathway | 10 | 0.025 (0.480) |  |
|  | Calcium signaling pathway) | 11 | 0.048 (0.660) |  |
| Bta-miR365-3p | Pathways in cancer (colorectal, prostate, non-small cell lung cancer, renal cell carcinoma, pancreatic cancer, small cell lung cancer, endometrical cancer) | 14 | 0.000 (0.045) | Cognitive function depression |
|  | Neutrophin signaling pathway | 9 | 0.001 (0.036) |  |
|  | mTOR signaling pathway | 6 | 0.001 (0.044) |  |
|  | Apoptosis | 7 | 0.003 (0.049) |  |
|  | Wnt signaling pathway) | 9 | 0.004 (0.043) |  |
|  | MAPK signaling pathway | 12 | 0.003 (0.039) |  |
|  | Chemokine signaling pathway | 9 | 0.009 (0.100) |  |
|  | Progesterone-mediated oocyte maturation | 6 | 0.022 (0.100) |  |
|  | Glioma | 5 | 0.017 (0.140) |  |
|  | Melanogenesis | 6 | 0.020 (0.150) |  |
|  | Regulation of actin cytoskeleton | 9 | 0.020 (0.140) |  |
|  | Long-term-potentiation | 5 | 0.023 (0.140) |  |
|  | Melanoma | 5 | 0.026 (0.150) |  |
|  | Chronic myeloid leukemia | 5 | 0.031 (0.160) |  |
|  | B cell receptor signaling pathway | 5 | 0.031 (0.160) |  |
|  | Vascular smooth muscle contraction | 6 | 0.032 (0.150) |  |
|  | Jak-STAT signaling pathway | 7 | 0.036 (0.160) |  |
|  | Cell cycle | 6 | 0.048 (0.200) |  |
| Bta-miR-499 | Pathways in cancer (colorectal cancer, basal cell carcinoma) | 11 | 0.021 (0.850) | - |
|  | Regulation of actin cytoskeleton | 8 | 0.038 (0.690) |  |
|  | Axon guidance | 6 | 0.041 (0.610) |  |
|  | Cell adhesion molecules | 6 | 0.044 (0.560) |  |
| Bta-miR-99b | Oocyte meiosis | 4 | 0.003 (0.130) | - |
|  | Pathways in cancer (colorectal cancer) | 5 | 0.010 (0.220) |  |
|  | Long-term potentiation | 3 | 0.014 (0.210) |  |
|  | Progesterone-mediated oocyte maturation | 3 | 0.023 (0.200) |  |
|  | Melanogenesis | 3 | 0.029 (0.210) |  |
| Bta-miR-24-3p | Axon guidance | 13 | 0.002 (0.210) | HIV, tuberculosis, cleft lip with cleft palate, cleft lip without cleft palate, arthritis, type 1 diabetes, recurrent pregnancy loss, paratyphoid feber, typhoid fever |
|  | MAPK signaling pathway | 19 | 0.005 (0.310) |  |
|  | Colorectal cancer | 9 | 0.009 (0.330) |  |
|  | Circadian rhythm | 4 | 0.009 ()0.380 |  |
|  | Melanogenesis | 9 | 0.022 (0.460) |  |
|  | Endocytosis | 13 | 0.027 (0.470) |  |
|  | Neurotrophin signaling pathway | 10 | 0.029 (0.440) |  |
|  | ErbB signaling pathway | 8 | 0.032 (0.430) |  |
|  | TGF-beta signaling pathway | 8 | 0.032 (0.430) |  |
|  | Regulation of actin cytoskeleton | 14 | 0.037 (0.440) |  |
|  | Wnt signaling pathway | 11 | 0.038 (0.410) |  |
|  | SNARE interactions in vesicular transport | 5 | 0.042 (0.420) |  |
|  | Calcium signaling pathway | 12 | 0.044 (0.40) |  |
|  | Focal adhesion | 13 | 0.048 (0.410) |  |
| Bta-miR-148a | Focal adhesion | 20 | 2.5E-4 (0.031) | Type 2 diabetes, cholesterol, atherosclerosis, endometriosis, height, juvenile arthritis, Huntington disease, Alzheimer’s disease |
|  | Glioma | 10 | 6.3E-4 (0.039) |  |
|  | Pathways in cancer (renal cell carcinoma, prostate cancer, pancreatic cancer, non-small cell lung cancer) | 26 | 7.6E-4 (0.032) |  |
|  | TGF-beta signaling pathway | 11 | 0.002 (0.055) |  |
|  | Regulation of actin cytoskeleton | 18 | 0.004 (0.090) |  |
|  | Hypertrophic cardiomyopathy | 10 | 0.005 (0.100) |  |
|  | Melanoma | 9 | 0.006 (0.087) |  |
|  | Wnt signaling pathway | 13 | 0.014 (0.160) |  |
|  | ECM-receptor interaction | 9 | 0.015 (0.160) |  |
|  | P53 signaling pathway | 8 | 0.015 (0.150) |  |
|  | ErbB signaling pathway | 9 | 0.019 (0.160) |  |
|  | Cell cycle | 11 | 0.022 (0.170) |  |
|  | Acute myeloid leukemia | 7 | 0.024 (0.170) |  |
|  | Chronic myeloid leukemia | 8 | 0.025 (0.170) |  |
|  | Dilated cardiomyopathy | 9 | 0.025 (0.160) |  |
|  | Axon guidance | 11 | 0.027 (0.170) |  |
|  | Adipocytokine signaling pathway | 7 | 0.044 (0.250) |  |
| Bta-miR-2339 | Adherens junction | 4 | 0.052 (0.990) | - |
|  | Axion guidance | 5 | 0.053 (0.910) |  |
| Bta-miR-23a | Pathways in cancer (renal cell carcinoma, colorectal cancer, pancreatic cancer, prostate cancer) | 45 | 1.1E-6 (1.6E-4) | IgE, spinocerebellar ataxia, Dupuytren’s disease, juvenile polyposis, cleft lip with cleft palate, cleft lip without cleft palate |
|  | Adherens junction | 18 | 4.5E-6 (3.2E-4) |  |
|  | Tight junction | 22 | 9.4E-5 (0.003) |  |
|  | Focal adhesion | 25 | 0.002 (0.053) |  |
|  | Regulation of actin cytoskeleton | 26 | 0.002 (0.052) |  |
|  | Glycosphingolipid biosynthesis | 7 | 0.004 (0.069) |  |
|  | MAPK signaling pathway | 29 | 0.006 (0.086) |  |
|  | FC gamma R-mediated phagocytosis | 14 | 0.007 (0.092) |  |
|  | Wnt signaling pathway | 19 | 0.007 (0.087) |  |
|  | Chronic myeloid leukemia | 12 | 0.007 (0.084) |  |
|  | Axon guidance  Chondroitin sulfate biosynthesis | 17 | 0.007 (0.078) |  |
|  | Endocytosis | 6 | 0.011 (0.110) |  |
|  | Neurotrophin signaling pathway | 15 | 0.025 (0.180) |  |
|  | P53signaling pathway | 10 | 0.027 (0.190) |  |
|  | Phosphatidylinositol signaling system | 10 | 0.044 (0.270) |  |
|  | TGF-beta signaling pathway | 11 | 0.049 (0.290) |  |
| Bta-miR21-3p | - | - | - | - |
| Bta-miR-486 | Melanoma | 6 | 8.6E-4 (0.066) | Post-traumatic stress disorder, Alzheimer’s disease, type 2 diabetes, autism, longevity, sporadic primary melanoma |
|  | Pathways in cancer (small cell lung cancer, prostate cancer) | 11 | 0.002 (0.078) |  |
|  | P53 signaling pathway | 5 | 0.006 (0.140) |  |
|  | Glioma | 4 | 0.029 (0.440) |  |
|  | Axon guidance | 5 | 0.047 (0.540) |  |
| Bta-miR-92a | Dilated cardiomyopathy | 11 | 0.006 (0.570) | Spinocerebellar ataxia |
|  | Hypertrophic cardiomyopathy | 10 | 0.011 (0.520) |  |
|  | Phosphatidylinositol signaling system | 9 | 0.014 (0.480) |  |
|  | Calcium signaling pathway | 15 | 0.019 (0.490) |  |
|  | Regulation of actin cytoskeleton | 17 | 0.023 (0.470) |  |
|  | Pantothenate and CoA biosynthesis | 4 | 0.025 (0.440) |  |
|  | Long-term potentiation | 8 | 0.027 (0.410) |  |
|  | Ubiquitin mediated proteolysis | 12 | 0.034 (0.450) |  |
|  | MAPK signaling pathway | 19 | 0.039 (0.460) |  |
|  | Cell cycle | 11 | 0.043 (0.460) |  |
| Bta-miR-184 | Chronic myeloid leukemia | 3 | 0.059 (0.761) | - |

*P-Value: The significance of gene-term enrichment with a modified Fisher's Exact Test

**Benjamini P-Value: Globally corrects enrichment p-values in order to control family-wide false discovery rate.
